# Management of Diabetic Gastroparesis

**DOI:** 10.4103/1319-3767.77237

**Published:** 2011

**Authors:** Badr M. Aljarallah

**Affiliations:** Department of Medicine, Gastroenterology Division, King Fahad Specialist Hospital, Faculty of Medicine, Qassim University, Maledia, Saudi Arabia

**Keywords:** Diabetes, gastroparesis, vomiting, gastric emptying, insulin, glucose

## Abstract

Symptoms suggestive of gastroparesis occur in 5% to 12% of patients with diabetes. Such a complication can affect both prognosis and management of the diabetes; therefore, practicing clinicians are challenged by the complex management of such cases. Gastroparesis is a disorder characterized by a delay in gastric emptying after a meal in the absence of a mechanical gastric outlet obstruction. This article is an evidence-based overview of current management strategies for diabetic gastroparesis. The cardinal symptoms of diabetic gastroparesis are nausea and vomiting. Gastroesophageal scintiscanning at 15-minute intervals for 4 hours after food intake is considered the gold standard for measuring gastric emptying. Retention of more than 10% of the meal after 4 hours is considered an abnormal result, for which a multidisciplinary management approach is required. Treatment should be tailored according to the severity of gastroparesis, and 25% to 68% of symptoms are controlled by prokinetic agents. Commonly prescribed prokinetics include metoclopramide, domperidone, and erythromycin. In addition, gastric electrical stimulation has been shown to improve symptoms, reduce hospitalizations, reduce the need for nutritional support, and improve quality of life in several open-label studies.

Gastroparesis is a syndrome characterized by delayed gastric emptying in the absence of mechanical obstruction of the stomach in patients with diabetes.[1] The cardinal symptoms include postprandial fullness, nausea, vomiting, and bloating.[2,3]

Patients with diabetes in whom gastroparesis develops often have had diabetes for at least 10 years. Symptoms attributed to gastroparesis occur in almost 5% to 12% of patients with diabetes.[4–6] The data suggest that delayed gastric emptying and its symptoms are generally stable during 12 years of follow-up or more.[7] In a study of 86 patients with diabetes who were followed-up for at least 9 years, gastroparesis was not associated with mortality after 10 adjustments for other disorders.[8] Diabetic patients with gastroparesis have a normal life expectancy after adjustment for other disorders.

## NORMAL GASTRIC EMPTYING

The proximal stomach serves as the reservoir for food, but also regulates the gastroduodenal flow rate and provides the space and time for pepsin and hydrochloric acid to initiate digestion.[9] Based on gastric barostat studies in human volunteers, over a liter of nutrients can be ingested without increased intragastric pressure.[10] Three main mechanisms involved in the regulation of the proximal stomach include the receptive relaxation reflex, the accommodation reflex, and enterogastric reflexes.[11] In the distal stomach, which acts as the grinder, there are 2 types of waves: the slow wave, controlled by the pacemaker cell (i.e., the interstitial cells of Cajal),[12] and phasic contractions, controlled by the migrating motor complex.[13] Neurohumoral factors play an essential role in the above mechanisms to control gastric motility.[14,15] The physical nature, particle size, fat, and caloric content of food determine its emptying rate. Solid nutrients usually empty in 2 phases over 3 to 4 hours.[16,17] An initial lag period (i.e., the retention phase) is followed by a propellant phase of relatively constant emptying. During the first phase, food gets churned[18] while antral contractions propel particles towards the closed pylorus. Foods are emptied once they have been broken down to particles of approximately 2 mm in diameter.

Liquid foods are usually emptied faster, especially in cases of large volumes. If there are increased calories in the liquid, emptying is relatively constant over time.

Neurohumoral factors include glucose regulating hormones, which are released when food arrives in different regions of the gut and play an essential role in the above mechanisms to control both gastric motility and postprandial glycemia.[19]

## GASTRIC EMPTYING AND ITS INFLUENCE ON BLOOD GLUCOSE AND INSULIN SECRETION

Nutrients empty from the stomach at an overall rate of about 2 to 3 kcal/min,[20] regulated predominantly by neural and hormonal feedback from the small intestine that further slows emptying by relaxing the fundus, suppressing antral and duodenal contractility, and stimulating tonic and phasic contractions.[21,22] Glucagon-like peptide-1 is one of the peptides involved in this feedback loop; others include glucose-dependent insulinotropic polypeptide, cholecystokinin, and peptide YY.[23] Although the rate of gastric emptying does not affect insulin secretion directly, it does regulate the delivery of carbohydrates and other macronutrients to the small intestine. This significantly impacts both the timing and magnitude of blood glucose excursion and peptide secretion, thereby modulating insulin release indirectly.

The effects of gastric emptying on both the peak and initial rise in blood glucose appear to be more direct than those of overall glycemia.[24] Similarly, the insulin requirement to sustain normoglycemia after a standard meal is substantially lower in type 1 diabetes patients with rather than without gastroparesis, the difference being apparent during the first 2 hours of the postprandial period.[25] Conversely, when gastric emptying is accelerated after ingestion of a meal with a smaller particle size in type 1 diabetes patients with gastroparesis, who are at risk for postprandial hypoglycemia, the postprandial blood glucose dip is diminished.[26]

In both healthy patients and type 2 diabetes patients, more rapid delivery of glucose to the small intestine does initially boost the peptide and insulin responses when compared with constant delivery of an identical glucose load. Nevertheless, this early increase in insulin cannot compensate for the greater initial rise in absorbed glucose, such that overall glycemic control is not improved.[27,28]

## CLINICAL DIAGNOSIS

Nausea and vomiting are the cardinal symptoms of gastroparesis in patients with diabetes, occurring in 92% and 84% of affected patients, respectively, along with bloating (75%) and early satiety (60%).[29,30] Only the most severe gastroparesis results in daily vomiting. The symptoms of gastroparesis also overlap with symptoms of metabolic disturbances, drug side effects, and other complications of diabetes (e.g., organic disorders, such as peptic ulcers, gastric and proximal small bowel obstruction, and biliary disease). Consequently, the diagnosis of gastroparesis is restricted to patients with chronic nausea and vomiting who have normal upper gastrointestinal imaging with objective evidence of profound delay in gastric emptying. Physical examination might be entirely normal, but in the most severe forms of gastroparesis dehydration and malnutrition might be present. Signs of associated systemic disease, such as systemic sclerosis, should be sought and particular care should be taken to ensure examination of the autonomic nervous system. An exaggerated postural drop both in systolic and diastolic blood pressure and on electrocardiogram might be a sign of autonomic neuropathy; vagal neuropathy is suspected if there is a loss of normal sinus dysrhythmia.[31]

Jones *et al*. concluded that the presence of abdominal bloating or fullness with no other upper gastrointestinal symptoms is associated with diabetic gastroparesis, and that gastric emptying is slower in diabetic women than in diabetic men.[32]

## DIAGNOSTIC TESTING

The gold standard test to diagnose gastroparesis is scintigraphy. However, other techniques are also available, such as ultrasonography, ^13^C breath testing, magnetic resonance imaging, swallowed capsule telemetry, antroduodenal manometry, and electrogastrography [Table 1].

**Table 1:** Types of diagnostic modality

Scintigraphy	Gold standard
^13^C breath test	Ideal alternative
Antroduodenal manometry	Distinguish between neuropathic and myopathic diseases
Electrogastrography	Focuses on the underlying myoelectrical activity
Swallowed capsule telemetry	Broadcasts in real time, intraluminal pH, phasic gastric pressure activity and ambient temperature
Magnetic resonance imaging	Measure gastric emptying and accommodation using sequential transaxial abdominal scans[104]

### Scintigraphy

In patients with suspected gastroparesis, ruling out obstruction is the first step, followed by determining the emptying time of the stomach. Scintiscanning at 15-minute intervals for 4 hours after food intake is considered the gold standard for measuring gastric emptying in detail. However, a simplified approach involving hourly scans to quantify residual gastric content is often used in practice.[19] The approach usually employs technetium-99 m (99mTc) sulphur colloid, bound or added to a uniform test meal. The caloric and nutritional content of the test meal should be standardized.[33,34] Retention of more than 10% of the meal after 4 hours is considered abnormal.[35] Compared with the gold standard approach, the simplified approach has a specificity of 62% and a sensitivity of 93%.[36] However, as it provides the actual percentage of food emptied and requires fewer scans, the simplified approach is generally preferred. Further, scintiscanning requires special equipment and expertise, and involves exposure to radiation, providing further reasons why the simplified method might be preferable. Certain points are important to consider when interpreting the results—the emptying rate for women is longer than for men, a daily variation in emptying rate of up to 20% is expected, and standards differ among laboratories.

### ^13^C breath test

Breath tests that measure gastric emptying involve ingestion of a meal enriched with a stable isotope, followed by the collection of breath samples; these are analyzed for carbon dioxide, using the isotope, at a reference laboratory. The profile of ^13^CO_2_ excretion is used to estimate the half-time of gastric emptying.[37] Compared with detailed scintiscanning, the breath test has a specificity of 80% and a sensitivity of 86%.[38] However, three studies have shown that the test correlates well with scintigraphy.[39–41] Significant lung disease and small bowel maldigestion and malabsorption might influence the test, and patients with lung, small bowel, pancreatic, and liver diseases are considered unsuitable candidates.[31]

### Antroduodenal manometry

Stationary or ambulatory antroduodenal manometry provides information on gastric and duodenal motor activity simultaneously. The information is helpful in building a full motor picture of the stomach and duodenum. Distinct patterns characterize the fasting and fed phases. Abnormal antroduodenal manometry has provided important information on the abnormalities occurring in diabetic gastroparesis, and it can distinguish between neuropathic and myopathic diseases. According to Mearin *et al*., abnormal antroduodenal manometry results have revealed tonic and phasic pylorospasms, as well as abnormal patterns of small intestine contractions.[42]

### Other screening measures

Other techniques are also available, such as electrogastrography, swallowed capsule telemetry, and magnetic resonance imaging. However, their roles in clinical practice still need to be further defined.

## MANAGEMENT

Managing patients with diabetic gastroparesis requires a multidisciplinary approach, and usually involves gastroenterologists, dietitians, diabetologists, social workers, and diabetes educators. An important preemptive strategy is prevention, which can be attempted by controlling glucose levels; this has been proposed in many trials of acute hyperglycemia.[43–48] Although there is a lack of clinical trials showing that the restoration of euglycemia or correction of electrolyte derangement normalizes gastric emptying or ameliorates symptoms, clinical experience and observational data suggest that improved metabolic control is beneficial in the prevention of gastroparesis in patients with diabetes. Further preemptive strategies involve the prevention of other exacerbating factors, such as electrolyte disturbances and improving nutritional status.

Some dietary modifications are appropriate, but the expected clinical benefits are often modest. Alcohol and smoking delay gastric emptying and these habits should be discouraged. Fat delays emptying and nondigestible fiber (found in fresh fruits and vegetables) might be poorly emptied, as both require effective interdigestive antral motility that is frequently absent in diabetic gastroparesis patients. Patients should be advised to consume a low-fat diet (without nondigestible fiber) and to take frequent, small meals–dietitians play an essential role in providing professional advice and following up with those patients. In more severe cases, substitution of mixed meals with homogenized or liquid meals supplemented with vitamins and minerals is required. Enteral nutrition via a jejunostomy tube might occasionally be required, while parenteral nutrition should be restricted to patients with severe gastric and small intestine dysmotility for whom enteral feeding is nearly impossible.

### Pharmacologic therapy

The aim of therapy is to improve the efficiency of the gastric pump and to relieve symptoms of nausea, bloating, vomiting, and pain. Selection of drugs is decided empirically. Two classes are available for treating affected patients, namely, prokinetic and antiemetic [Table 2].

**Table 2:** Management options

Pharmacologic therapy	
Prokinetic agents	Little evidence from clinical trials to support the use of specific prokinetic regimens
Antiemetic agents	
Botulinum toxins	Restricted to clinical trials
Gastric electrical stimulation	Further studies are needed to define the best stimulation strategy and the mechanisms responsible for clinical improvement
Surgery	Insufficient data
Acupuncture	Insufficient data

#### Prokinetic agents

This class of drugs consists of the most commonly used medications for gastroparesis. Prokinetics stimulate peristalsis and might specifically improve gastric pump function by influencing antral contractility and rhythm as well as antroduodenal coordination.

Clinical trials have shown that compared with placebo these agents have increased gastric emptying by about 25%–72% and have reduced the severity of symptoms (typically measured with the use of Likert scales) by 25%–68%.[3,49–59] However, many of these trials were small, some were not blinded, some included patients with gastroparesis owing to causes other than diabetes, and many correlated poorly with influence on gastric emptying.[60,61]

Patterson and colleagues[53] conducted a double-blind, multicentre comparison of domperidone and metaclopramide for the treatment of patients with diabetes and concluded that each effectively reduced the symptoms of diabetic gastroparesis; however, central nervous system (CNS) side effects were more pronounced in patients taking metaclopramide. These side effects are believed to be more prevalent after long-term use, although data are conflicting and incomplete.[62–64]

One review looking at continuous use of metaclopramide showed insufficient evidence to conclude whether metaclopramide is efficacious for chronic use; routine monitoring might mitigate any risk associated with metaclopramide therapy.[65]

Domperidone is also widely used in this condition. Twenty-eight trials have assessed the efficacy of domperidone in diabetic gastroparesis, with a combined sample size of 1016 patients.[56,57,66–71] Overall, 64% of the studies showed significant improvement of symptoms with domperidone, 60% showed an efficacy in gastric emptying, and 67% proved the drug to be effective in reducing hospital admissions. These results need to be interpreted very cautiously, owing to the substantial methodological limitations of these studies. Unlike metaclopramide, domperidone does not cross the blood brain barrier; hence, there are no CNS side effects.

Cisapride, another prokinetic drug option, is associated with an increased risk of cardiac arrhythmia, including torsades de pointes, and is therefore currently withdrawn from the market and any future use requires special authorization.

Erythromycin is a motilin agonist that greatly increases the fractional rate of gastric emptying. Although a number of studies document the efficacy of erythromycin in improving gastric emptying, little information exists concerning symptom improvement in patients with diabetic gastroparesis. Thirty-five clinical trials[72] were conducted, and three studies[58,73,74] evaluated patients with diabetic gastroparesis (comprising a total number of 29 diabetic patients). None of the trials used symptoms as a primary end point, but instead studied different dose regimens and follow-up periods (e.g., 2 to 4 weeks). In one of these studies,[73] the average half-time of gastric emptying in diabetic subjects was 110 minutes (range 77 to 120) before treatment. The half-time decreased to an average of 55 minutes (range 29 to 15) after 3 weeks of treatment with erythromycin. Unlike in its oral form, intravenous erythromycin (3 mg/kg of body weight every 8 hours by infusion over 45 minutes) is more effective than placebo in relieving acute nondiabetic gastroparesis in hospitalized patients; however, no trials have included patients with diabetes or compared erythromycin with another agent.

Tegaserod is used in the treatment of women with constipation and predominant irritable bowel syndrome. The drug stimulates small intestine motility and, in healthy volunteers, there is evidence both for and against gastric prokinetic activity. The potential to influence gastric pump activity has been used to recommend its trial in gastroparesis, but currently there is no good evidence to support its use. Also, physicians require special authorization to prescribe tegaserod in clinical practice.

In summary, there is little evidence from clinical trials to support the use of specific prokinetic regimens in gastroparesis, but clinical experience suggests that a range of prokinetic drugs might be helpful in controlling nausea and vomiting. The choice of prokinetic is empirical, but it is common practice to initially prescribe metaclopramide (10 mg, 1 to 3 times daily) or domperidone (10 mg, 1 to 3 times daily). If both of these fail to control symptoms, erythromycin (250 mg, 3 times daily) should be the next option.

#### Antiemetic agents

Phenothiazines and antihistamines are commonly prescribed agents. Antiemetics are helpful in relieving vomiting. There are few clinical trials to support the use of specific agents. However, it is common practice to start patients with prochlorperazine and chlorpromazine; 5-hydroxytryptamine 3 receptor antagonists might be prescribed if these prove to be ineffective. In choosing which agents to use, availability and side effects should be considered.

### Botulinum toxins

Botulinum toxins have been used in clinical trials on account of observational evidence of pylorospasms in manometric studies of patients with diabetes. *Pylorospasms* can be defined as prolonged periods of increased pyloric tone and phasic contractions. Botulinum toxins inhibit these contractions and have been proposed to improve symptoms. Data from a single-institution, randomized, double-blind, placebo-controlled trial[75] involving 16 patients with gastroparesis (only 9 of whom had diabetes) showed that botulinum toxin is not superior to placebo after 1 month of treatment. In concordance with those results, an earlier controlled trial also showed no efficacy.[76] However, four previous uncontrolled studies have suggested that endoscopic injections of botulinum toxins are efficacious.[77–80] One of the important points raised in the above studies is whether improvement of gastroparesis would necessarily improve the symptoms; the answer to that is not yet clear. Until a well-controlled study addressing botulinum toxin injections in patients with diabetic gastroparesis takes place, such a treatment should be restricted to clinical trials.

### Gastric electrical stimulation

Gastric electrical stimulation (GES) involves the use of surgically placed electrodes in the muscle wall of the stomach cavity, about 9.5 to 10 cm from the pylorus along the greater curvature of the antrum.[81] The two electrodes are tangentially placed 1 cm apart deep in the muscularis propria, and are connected to a neurostimulator in a pocket of the abdominal wall. Intraoperative endoscopy is performed at the time of implantation to ensure that the leads are not accidentally pushed into the lumen of the stomach and are positioned appropriately in the muscularis propria of the stomach wall. The stimulator is set to generate high frequency (12 cycles per minute) low energy pulses (300 microsecond pulse width, 4 to 5 mA) for a short duration. In several open-label studies with mean follow-up periods of 3.7 to 4.3 years, short-pulse stimulation improved symptoms (in particular nausea and vomiting), reduced hospitalizations, reduced the need for nutritional support, and improved quality of life.[81–89] The first double-blind crossover trial[90] was published in 2003 and involved 33 patients with idiopathic or diabetic gastroparesis; each patient had 1 month on GES, 1 month off GES, and follow-up after 12 months. Patients included in the study had symptoms of gastroparesis for more than 1 year, were drug refractory, and had a vomiting frequency of more than 7 times per week. Overall, 79% of patients had a 55% decrease in vomiting only. Among the 17 patients in the study with diabetes, the median frequency of episodes of vomiting per week was 6.0 with the stimulator on and 12.8 with the stimulator off (*P*=0.16). In most of these studies, GES had no effect on gastric emptying; hence, the mechanism by which electrical stimulation improves symptoms is still unclear. GES has been proven to modulate the CNS in animal studies.[91] However, in a double-blind study from Denmark of 7 diabetes patients with medically refractory gastroparesis, no evidence was found for GES-induced modulation of the visceral sensory system and central excitability.[92]

Long-pulse GES has been reported to normalize slow-wave dysrhythmia and improve gastric emptying in patients with gastroparesis.[93] However, no implantable pulse generator is available to deliver long-pulse stimuli.

The most commonly seen complication has involved pacemakers, in which hardware infection has occurred requiring removal of the device.

Beyond clinical trials, there is insufficient sham-controlled evidence to support this therapy at the current time; further studies are needed to define the best stimulation strategy and the mechanisms responsible for clinical improvement.

### Surgery

A systematic review[94] identified three uncontrolled studies[95–97] involving 32 patients using jejunostomy tubes with modest improvement of symptoms and nutrition intake. Also identified were three small case series[98–100] involving 6 patients who underwent pyloroplasty and gastrectomy, with minimal overall improvement. As such, the data for surgical treatment are insufficient to provide support for gastric surgical procedures in the treatment of patients with diabetic gastroparesis.

### Acupuncture

Two studies assessed the effects of acupuncture in patients with diabetic gastroparesis.[101,102] In the study by Wang *et al*.,[101] individual acupuncture was superior to drug treatment (i.e., domperidone) with respect to symptoms, with a total efficacy rate of 94%. However, placebo control, blinding of patients or providers, statistical analyses, and calculations for effective rates were not described in the publication. In the uncontrolled study by Chang *et al*.,[102] cutaneous electrogastrography and serum parameters after needling of acupressure point ST-36 were assessed in 15 patients with diabetic gastroparesis. This experimental design revealed significant changes in electrogastrography and serum parameters after acupuncture, but presented no information about clinical effects.[103]

## RECOMMENDATIONS

The purpose of this article is to increase awareness of diabetic gastroparesis and its complications for both physicians and patients. Recommendations based on the aforementioned findings are as follows:

- Any patient with diabetes who presents with recurrent nausea and vomiting should undergo scintigraphy to diagnose gastroparesis after first ruling out a gastric obstruction.
- Optimal blood sugar control, in consultation with a dietician, is a necessary first step.
- Treatment should be tailored according to the severity of gastroparesis, beginning with a combination of prokinetic and antiemetic drugs (metaclopramide, 10 mg 3 times daily before meals, and/either 10 mg prochlorperazine or 50 mg dimenhydrinate every 12 hours).
- If oral feeding is impossible, a trial of enteral feeding or parenteral feeding should be considered.
